# (η^5^-Cyclo­penta­dien­yl)(η^6^-mesitylamine)ruthenium(II) hexa­fluorido­phosphate

**DOI:** 10.1107/S1600536809016894

**Published:** 2009-05-14

**Authors:** Eva Becker, Karl Kirchner, Kurt Mereiter

**Affiliations:** a Institute of Applied Synthetic Chemistry, Vienna University of Technology, Getreidemarkt 9/163, A-1060 Vienna, Austria; b Institute of Chemical Technologies and Analytics, Vienna University of Technology, Getreidemarkt 9/164SC, A-1060 Vienna, Austria

## Abstract

The title compound, [Ru(η^5^-C_5_H_5_){η^6^-C_6_H_2_(CH_3_)_3_NH_2_}]PF_6_, contains a sandwich complex with a mesitylamine unit which is significantly non-planar at the *ipso*-carbon of the amino group due to repulsive electronic effects with Ru. The *ipso*-carbon deviates by 0.107 (3) Å from the least-squares plane of the remaining five benzene ring atoms, which show an r.m.s. deviation of 0.005 Å. N—H⋯F hydrogen-bonding interactions help to consolidate the crystal packing.

## Related literature

For general background and a related structure with —N(CH_3_)_2_ instead of —NH_2_, see: Standfest-Hauser *et al.* (2003 ▶). For related chromium arene complexes, see: Djukic *et al.* (2000 ▶); Hunter *et al.* (1992 ▶). For synthetic details, see: Gill & Mann (1982 ▶); Kündig & Monnier (2004 ▶).
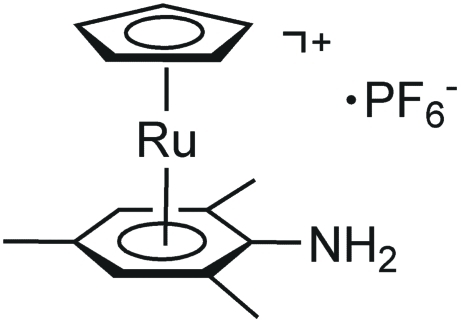


## Experimental

### 

#### Crystal data

[Ru(C_5_H_5_)(C_9_H_13_N)]PF_6_
*M* *_r_* = 446.33
Orthorhombic, 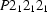

*a* = 7.5119 (4) Å
*b* = 10.2047 (6) Å
*c* = 21.1818 (12) Å
*V* = 1623.73 (16) Å^3^
*Z* = 4
Mo *K*α radiation
μ = 1.12 mm^−1^
*T* = 173 K
0.55 × 0.30 × 0.26 mm

#### Data collection

Bruker SMART CCD diffractometer
Absorption correction: multi-scan (*SADABS*; Bruker, 2003 ▶) *T*_min_ = 0.62, *T*_max_ = 0.75
24305 measured reflections
4741 independent reflections
4650 reflections with *I* > 2σ(*I*)
*R*_int_ = 0.026

#### Refinement

*R*[*F* ^2^ > 2σ(*F* ^2^)] = 0.024
*wR*(*F* ^2^) = 0.055
*S* = 1.12
4741 reflections
218 parameters
1 restraint
H atoms treated by a mixture of independent and constrained refinement
Δρ_max_ = 0.65 e Å^−3^
Δρ_min_ = −0.41 e Å^−3^
Absolute structure: Flack (1983 ▶), 2202 Friedel pairs
Flack parameter: 0.21 (3)

### 

Data collection: *SMART* (Bruker, 2003 ▶); cell refinement: *SAINT* (Bruker, 2003 ▶); data reduction: *SAINT*; program(s) used to solve structure: *SHELXS97* (Sheldrick, 2008 ▶); program(s) used to refine structure: *SHELXL97* (Sheldrick, 2008 ▶); molecular graphics: *SHELXTL* (Sheldrick, 2008 ▶); software used to prepare material for publication: *SHELXTL*.

## Supplementary Material

Crystal structure: contains datablocks global, I. DOI: 10.1107/S1600536809016894/gk2208sup1.cif

Structure factors: contains datablocks I. DOI: 10.1107/S1600536809016894/gk2208Isup2.hkl

Additional supplementary materials:  crystallographic information; 3D view; checkCIF report

## Figures and Tables

**Table 1:** Selected bond lengths (Å)

Ru—C1	2.179 (2)
Ru—C2	2.164 (2)
Ru—C3	2.179 (3)
Ru—C4	2.181 (3)
Ru—C5	2.187 (3)
Ru—C6	2.314 (2)
Ru—C7	2.212 (2)
Ru—C8	2.178 (2)
Ru—C9	2.214 (2)
Ru—C10	2.185 (2)
Ru—C11	2.229 (2)

**Table 2:** Hydrogen-bond geometry (Å, °)

*D*—H⋯*A*	*D*—H	H⋯*A*	*D*⋯*A*	*D*—H⋯*A*
N—H1*A*⋯F1	0.87 (2)	2.26 (2)	3.106 (3)	163 (3)
N—H1*B*⋯F5^i^	0.87 (2)	2.43 (3)	3.174 (3)	143 (3)

Symmetry code: (i) 
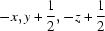
.

**Fig. 1.**
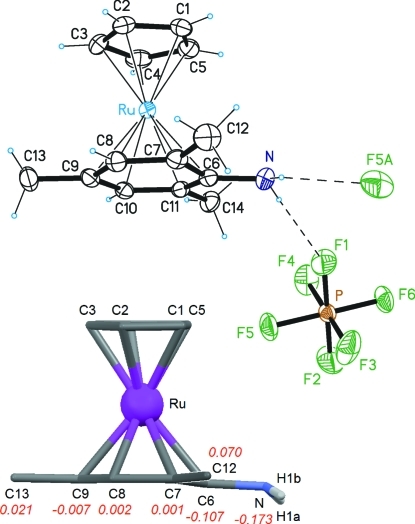
Perspective view of (I). Displacement ellipsoids shown at the 40% probability level. The sideview of the Ru complex on the lower left depicts the out-of-plane displacements of C6 and N. The red numbers give the deviations (Å) of the respective atoms from the least-squares plane C7—C8—C9—C10—C11. F5A corresponds to F5i of Table 2.

**Fig. 2.**
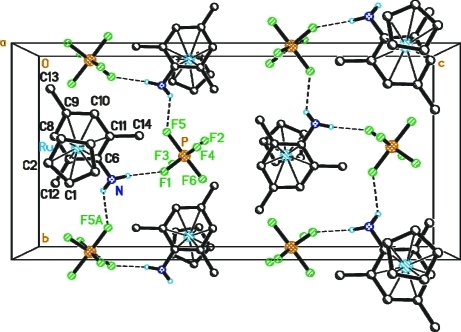
Packing diagram of (I), viewed down a, with N—H···F bonds as dashed lines. Carbon-bound H atoms omitted.

## supplementary crystallographic information

### Comment

We have shown (Standfest-Hauser *et al.*, 2003) that arene amines hapto-6-coordinated to a cyclopentadienylruthenium fragment (CpRu) are not planar but display a significant shift of the *ipso*-carbon bearing the amino substituent out of the mean aromatic plane away from the CpRu fragment by about 0.1 to 0.2 Å. This corresponds to a envelope-type folding of the ring by 7–15° for the interplanar angle. The same effect was previously reported for chromium arene complexes (Hunter *et al.*, 1992; Djukic, *et al.*, 2000). Recently, we obtained the title compound, (I), in a crystalline form. This offered the opportunity to study the mentioned effect for a compound with NH_2_ instead of N(CH_3_)_2_. In (I) the cyclopentadienyl ring and the 5-membered ring segment C7—C8—C9—C10—C11 are almost perfectly planar (r.m.s. aplanarities 0.006 and 0.005 Å, respectively) and mutually inclined by 1.0 (1)° (Fig. 1). The *ipso*-carbon C6 and the amino nitrogen deviate by 0.107 (3) and 0.172 (4) Å, respectively, from ring segment plane and both are bent away from the CpRu fragment. Thus the envelope-type ring folding angle, measured between planes C7—C6—C11 and C7—C8—C9—C10—C11, is 8.3 (3)°. According to FT/B3LYP calculations (Standfest-Hauser *et al.*, 2003) the reason for this envelope deformation of the benzene ring is that the surplus of π-electron density at C6 arising from the π-donor substituent NH_2_ becomes less pronounced and a 8° folding was predicted for free [CpRu(η^6^-C_6_H_5_NH_2_)]^+^. These quantities are comparable with the experimental data of [CpRu(η^6^-C_6_H_5_N(CH_3_)_2_)]PF_6_ (Standfest-Hauser *et al.*, 2003), 0.125 (3) Å (deviation of the *ipso*-C from the plane of the remaining ring atoms) and 10° (envelope-type ring folding angle), respectively. In this compound and its 2-dimethylamino-pyridine congener, the N-bound methyl groups are bent toward the CpRu moieties due to predicted orbital repulsion effects between dimethylamino nitrogen and *ipso*-carbon. This differs from (I), where the hydrogen atoms are bent off from the CpRu moiety and the nitrogen behaves more pyramidal. We attribute this deviation to the formation of two N—H···F hydrogen bonds (Fig. 1 and Table 2), absent in the dimethylamino compounds. As shown in Fig. 2, these bonds form zigzag chains along the *b* axis of (I). Further structural coherence is provided by π-π-stacking between Ru complexes, which form columns along the *a* axis with short stacking distances such as C4—C6(1 + *x*,*y*,*z*) = 3.482 (4) Å and C3—C7(1 + *x*,*y*,*z*) = 3.572 (4) Å. Weak C—H···F interactions donated by methyl, Cp and arene H-atoms stiffen the structure too, *e.g.* C3···F6(1 - *x*,-1/2 + *y*,1/2 - *z*) = 3.280 (4) Å.

### Experimental

To a solution of [CpRu(CH_3_CN)_3_]PF_6_ (Gill, & Mann, 1982; Kündig & Monnier, 2004; 120 mg, 0.276 mmol) in CH_2_Cl_2_ (5 ml) mesitylamine (41 µ*L*, 0.290 mmol) was added. After the mixture was stirred at room temperature for 1 h, the solvent was removed under vacuum and the resulting white solid of (I) was collected on a glass frit and washed twice with diethyl ether (10 ml). Yield: 118 mg (96%). ^1^H NMR (δ, acetone-d_6_, 20°C): 6.02 (s, 2H, Mes^3,5^), 5.13 (bs, 2H, NH_2_), 5.09 (s, 5H, Cp), 2.31 (s, 6H, Me^2,6^), 2.17 (s, 3H, Me^4^). ^13^C{^1^H} NMR (δ, acetone-d_6_, 20°C): 123.3 (1 C, Mes^1^), 94.9 (1 C, Mes^4^), 87.0 (2 C, Mes^2,6^), 83.7 (2 C, Mes^3,5^), 79.9 (5 C, Cp), 18.5 (1 C, Me^4^), 16.8 (2 C, Me^2,6^). Colourless crystals of (I) were grown from CH_2_Cl_2_ using vapour diffusion of diethyl ether at room temperature.

### Refinement

Refinement of the Flack (1983) parameter with 2202 Friedel pairs led to a value of 0.21 (3); the crystal was thus assumed to be an inversion twin with unequal components and a corresponding twin scale factor was applied. All C-bound H atoms were placed in calculated positions and thereafter treated as riding. A torsional parameter was refined for each methyl group. The two nitrogen bound H atoms were refined in *x*,*y*,*z* restraining both N—H bonds to be identical in lengths. *U*_iso_(H) = 1.2*U*_eq_(C_arene_,*N*) and *U*_iso_(H) = 1.5*U*_eq_(C_methyl_) were used.

### Crystal data

**Table:**

[Ru(C_5_H_5_)(C_9_H_13_N)]F_6_P	*F*(000) = 888
*M**_r_* = 446.33	*D*_x_ = 1.826 Mg m^−^^3^
Orthorhombic, *P*2_1_2_1_2_1_	Mo *K*α radiation, λ = 0.71073 Å
Hall symbol: P 2ac 2ab	Cell parameters from 7583 reflections
*a* = 7.5119 (4) Å	θ = 2.2–30.0°
*b* = 10.2047 (6) Å	µ = 1.12 mm^−^^1^
*c* = 21.1818 (12) Å	*T* = 173 K
*V* = 1623.73 (16) Å^3^	Prism, colourless
*Z* = 4	0.55 × 0.30 × 0.26 mm

### Data collection

**Table:**

Bruker SMART CCD diffractometer	4741 independent reflections
Radiation source: fine-focus sealed tube	4650 reflections with *I* > 2σ(*I*)
graphite	*R*_int_ = 0.026
ω scans	θ_max_ = 30.0°, θ_min_ = 2.2°
Absorption correction: multi-scan (*SADABS*; Bruker, 2003)	*h* = −10→10
*T*_min_ = 0.62, *T*_max_ = 0.75	*k* = −14→14
24305 measured reflections	*l* = −29→29

### Refinement

**Table:**

Refinement on *F*^2^	Secondary atom site location: difference Fourier map
Least-squares matrix: full	Hydrogen site location: inferred from neighbouring sites
*R*[*F*^2^ > 2σ(*F*^2^)] = 0.024	H atoms treated by a mixture of independent and constrained refinement
*wR*(*F*^2^) = 0.055	*w* = 1/[σ^2^(*F*_o_^2^) + (0.0228*P*)^2^ + 0.8717*P*] where *P* = (*F*_o_^2^ + 2*F*_c_^2^)/3
*S* = 1.12	(Δ/σ)_max_ = 0.001
4741 reflections	Δρ_max_ = 0.65 e Å^−^^3^
218 parameters	Δρ_min_ = −0.41 e Å^−^^3^
1 restraint	Absolute structure: Flack, (1983), 2202 Friedel pairs
Primary atom site location: structure-invariant direct methods	Flack parameter: 0.21 (3)

### Special details

**Table:**

Geometry. All e.s.d.'s (except the e.s.d. in the dihedral angle between two l.s. planes) are estimated using the full covariance matrix. The cell e.s.d.'s are taken into account individually in the estimation of e.s.d.'s in distances, angles and torsion angles; correlations between e.s.d.'s in cell parameters are only used when they are defined by crystal symmetry. An approximate (isotropic) treatment of cell e.s.d.'s is used for estimating e.s.d.'s involving l.s. planes.
Refinement. Refinement of *F*^2^ against ALL reflections. The weighted *R*-factor *wR* and goodness of fit *S* are based on *F*^2^, conventional *R*-factors *R* are based on *F*, with *F* set to zero for negative *F*^2^. The threshold expression of *F*^2^ > σ(*F*^2^) is used only for calculating *R*-factors(gt) *etc*. and is not relevant to the choice of reflections for refinement. *R*-factors based on *F*^2^ are statistically about twice as large as those based on *F*, and *R*- factors based on ALL data will be even larger.

### Fractional atomic coordinates and isotropic or equivalent isotropic displacement parameters (Å^2^)

**Table:**

	*x*	*y*	*z*	*U*_iso_*/*U*_eq_	
Ru	0.62135 (2)	0.480064 (16)	0.128280 (7)	0.02275 (4)	
C1	0.7945 (3)	0.6499 (2)	0.11758 (13)	0.0375 (5)	
H1	0.7526	0.7358	0.1088	0.045*	
C2	0.8399 (3)	0.5545 (3)	0.07219 (14)	0.0401 (6)	
H2	0.8323	0.5645	0.0277	0.048*	
C3	0.8987 (4)	0.4415 (3)	0.10452 (14)	0.0431 (6)	
H3	0.9400	0.3629	0.0855	0.052*	
C4	0.8854 (4)	0.4655 (3)	0.17054 (13)	0.0427 (6)	
H4	0.9136	0.4057	0.2034	0.051*	
C5	0.8217 (3)	0.5963 (3)	0.17799 (13)	0.0388 (6)	
H5	0.8012	0.6397	0.2170	0.047*	
N	0.2758 (3)	0.6420 (2)	0.19495 (11)	0.0374 (5)	
H1A	0.237 (4)	0.625 (3)	0.2330 (10)	0.045*	
H1B	0.219 (4)	0.699 (3)	0.1717 (13)	0.045*	
C6	0.3383 (3)	0.5357 (2)	0.16198 (11)	0.0284 (4)	
C7	0.3543 (3)	0.5395 (2)	0.09480 (11)	0.0293 (5)	
C8	0.4115 (3)	0.4252 (2)	0.06244 (11)	0.0308 (5)	
H8	0.4138	0.4263	0.0176	0.037*	
C9	0.4649 (3)	0.3105 (2)	0.09396 (11)	0.0299 (5)	
C10	0.4628 (3)	0.3137 (2)	0.16078 (11)	0.0278 (4)	
H10	0.5012	0.2384	0.1833	0.033*	
C11	0.4056 (3)	0.4246 (2)	0.19536 (10)	0.0265 (4)	
C12	0.3062 (4)	0.6610 (3)	0.05839 (14)	0.0426 (6)	
H12A	0.1773	0.6752	0.0607	0.064*	
H12B	0.3416	0.6503	0.0142	0.064*	
H12C	0.3683	0.7366	0.0765	0.064*	
C13	0.5281 (4)	0.1920 (3)	0.05904 (13)	0.0418 (6)	
H13A	0.4266	0.1347	0.0500	0.063*	
H13B	0.6149	0.1446	0.0849	0.063*	
H13C	0.5840	0.2190	0.0193	0.063*	
C14	0.4080 (4)	0.4215 (3)	0.26627 (10)	0.0373 (5)	
H14A	0.2864	0.4101	0.2821	0.056*	
H14B	0.4570	0.5041	0.2823	0.056*	
H14C	0.4822	0.3484	0.2806	0.056*	
P	−0.05498 (8)	0.52386 (6)	0.36805 (3)	0.02999 (11)	
F1	0.0612 (3)	0.6046 (2)	0.31902 (9)	0.0629 (6)	
F2	−0.1653 (4)	0.4427 (2)	0.41838 (11)	0.0828 (8)	
F3	−0.2304 (3)	0.5675 (2)	0.33379 (11)	0.0746 (7)	
F4	0.1256 (3)	0.4786 (2)	0.40177 (8)	0.0631 (5)	
F5	−0.0414 (3)	0.39806 (18)	0.32320 (9)	0.0553 (5)	
F6	−0.0659 (3)	0.64704 (17)	0.41381 (8)	0.0499 (4)	

### Atomic displacement parameters (Å^2^)

**Table:**

	*U*^11^	*U*^22^	*U*^33^	*U*^12^	*U*^13^	*U*^23^
Ru	0.02249 (6)	0.02258 (7)	0.02320 (7)	−0.00058 (6)	0.00164 (6)	−0.00027 (6)
C1	0.0313 (11)	0.0299 (11)	0.0513 (16)	−0.0078 (9)	0.0047 (11)	−0.0006 (10)
C2	0.0343 (13)	0.0432 (15)	0.0427 (14)	−0.0109 (10)	0.0131 (10)	−0.0033 (11)
C3	0.0276 (12)	0.0408 (13)	0.0611 (16)	0.0041 (10)	0.0135 (11)	−0.0093 (11)
C4	0.0251 (10)	0.0511 (16)	0.0518 (14)	−0.0046 (14)	−0.0064 (11)	0.0103 (12)
C5	0.0288 (11)	0.0484 (16)	0.0390 (13)	−0.0084 (10)	−0.0021 (10)	−0.0113 (12)
N	0.0328 (11)	0.0345 (11)	0.0448 (13)	0.0059 (9)	0.0059 (9)	−0.0055 (9)
C6	0.0225 (9)	0.0272 (11)	0.0355 (11)	−0.0014 (8)	0.0028 (7)	−0.0007 (9)
C7	0.0233 (10)	0.0301 (12)	0.0345 (11)	0.0011 (9)	−0.0039 (8)	0.0036 (8)
C8	0.0309 (12)	0.0350 (11)	0.0264 (10)	−0.0030 (9)	−0.0019 (8)	−0.0008 (9)
C9	0.0309 (12)	0.0277 (11)	0.0310 (11)	−0.0055 (9)	−0.0006 (9)	−0.0031 (9)
C10	0.0296 (11)	0.0227 (10)	0.0309 (11)	−0.0029 (9)	0.0028 (9)	0.0036 (8)
C11	0.0226 (10)	0.0294 (10)	0.0275 (10)	−0.0026 (8)	0.0025 (8)	−0.0007 (8)
C12	0.0407 (14)	0.0400 (14)	0.0472 (15)	0.0078 (12)	−0.0066 (12)	0.0126 (12)
C13	0.0531 (16)	0.0315 (13)	0.0407 (14)	−0.0017 (12)	0.0040 (12)	−0.0098 (10)
C14	0.0424 (14)	0.0413 (13)	0.0282 (11)	0.0009 (11)	0.0072 (9)	0.0003 (9)
P	0.0352 (3)	0.0283 (2)	0.0265 (2)	−0.0024 (2)	0.0010 (2)	−0.0034 (3)
F1	0.0797 (14)	0.0524 (11)	0.0566 (11)	−0.0027 (10)	0.0338 (10)	0.0086 (9)
F2	0.113 (2)	0.0541 (12)	0.0812 (14)	−0.0239 (12)	0.0484 (15)	0.0043 (10)
F3	0.0539 (11)	0.0992 (17)	0.0708 (13)	0.0344 (12)	−0.0239 (10)	−0.0324 (12)
F4	0.0666 (11)	0.0686 (12)	0.0541 (10)	0.0186 (13)	−0.0253 (9)	−0.0099 (9)
F5	0.0627 (11)	0.0458 (10)	0.0574 (11)	0.0050 (9)	−0.0066 (9)	−0.0271 (9)
F6	0.0722 (12)	0.0392 (8)	0.0381 (8)	−0.0013 (8)	0.0039 (8)	−0.0153 (7)

### Geometric parameters (Å, °)

**Table:**

Ru—C1	2.179 (2)	C4—C5	1.427 (4)
Ru—C2	2.164 (2)	C4—H4	0.9500
Ru—C3	2.179 (3)	C5—H5	0.9500
Ru—C4	2.181 (3)	N—H1A	0.87 (2)
Ru—C5	2.187 (3)	N—H1B	0.87 (2)
Ru—C6	2.314 (2)	C7—C12	1.505 (3)
Ru—C7	2.212 (2)	C8—H8	0.9500
Ru—C8	2.178 (2)	C9—C13	1.495 (3)
Ru—C9	2.214 (2)	C10—H10	0.9500
Ru—C10	2.185 (2)	C11—C14	1.503 (3)
Ru—C11	2.229 (2)	C12—H12A	0.9800
C6—N	1.373 (3)	C12—H12B	0.9800
C6—C7	1.429 (3)	C12—H12C	0.9800
C6—C11	1.428 (3)	C13—H13A	0.9800
C7—C8	1.419 (3)	C13—H13B	0.9800
C11—C10	1.415 (3)	C13—H13C	0.9800
C8—C9	1.406 (3)	C14—H14A	0.9800
C10—C9	1.416 (3)	C14—H14B	0.9800
C1—C5	1.406 (4)	C14—H14C	0.9800
C1—C2	1.410 (4)	P—F3	1.569 (2)
C1—H1	0.9500	P—F2	1.584 (2)
C2—C3	1.412 (4)	P—F1	1.5876 (18)
C2—H2	0.9500	P—F6	1.5894 (16)
C3—C4	1.423 (4)	P—F5	1.6002 (17)
C3—H3	0.9500	P—F4	1.6014 (19)
			
C2—Ru—C8	106.72 (10)	C1—C5—C4	108.2 (2)
C2—Ru—C1	37.90 (10)	C1—C5—Ru	70.90 (14)
C8—Ru—C1	124.74 (10)	C4—C5—Ru	70.72 (16)
C2—Ru—C3	37.94 (11)	C1—C5—H5	125.9
C8—Ru—C3	119.84 (10)	C4—C5—H5	125.9
C1—Ru—C3	63.19 (10)	Ru—C5—H5	124.1
C2—Ru—C4	63.86 (11)	C6—N—H1A	115 (2)
C8—Ru—C4	154.62 (10)	C6—N—H1B	114 (2)
C1—Ru—C4	63.52 (10)	H1A—N—H1B	120 (3)
C3—Ru—C4	38.10 (11)	N—C6—C11	119.8 (2)
C2—Ru—C10	149.22 (10)	N—C6—C7	120.9 (2)
C8—Ru—C10	66.89 (9)	C11—C6—C7	119.0 (2)
C1—Ru—C10	167.48 (9)	N—C6—Ru	131.66 (17)
C3—Ru—C10	116.98 (10)	C11—C6—Ru	68.45 (12)
C4—Ru—C10	108.27 (10)	C7—C6—Ru	67.78 (13)
C2—Ru—C5	63.38 (10)	C8—C7—C6	119.0 (2)
C8—Ru—C5	161.08 (10)	C8—C7—C12	120.2 (2)
C1—Ru—C5	37.59 (10)	C6—C7—C12	120.9 (2)
C3—Ru—C5	63.34 (10)	C8—C7—Ru	69.83 (13)
C4—Ru—C5	38.14 (11)	C6—C7—Ru	75.51 (13)
C10—Ru—C5	130.17 (10)	C12—C7—Ru	127.45 (17)
C2—Ru—C7	114.56 (11)	C9—C8—C7	122.7 (2)
C8—Ru—C7	37.71 (9)	C9—C8—Ru	72.73 (13)
C1—Ru—C7	106.85 (9)	C7—C8—Ru	72.46 (13)
C3—Ru—C7	147.39 (11)	C9—C8—H8	118.6
C4—Ru—C7	167.10 (10)	C7—C8—H8	118.6
C10—Ru—C7	79.60 (9)	Ru—C8—H8	128.6
C5—Ru—C7	129.03 (10)	C8—C9—C10	116.9 (2)
C2—Ru—C9	119.77 (10)	C8—C9—C13	121.9 (2)
C8—Ru—C9	37.34 (9)	C10—C9—C13	121.1 (2)
C1—Ru—C9	154.73 (10)	C8—C9—Ru	69.93 (13)
C3—Ru—C9	106.88 (10)	C10—C9—Ru	70.12 (14)
C4—Ru—C9	124.33 (10)	C13—C9—Ru	128.78 (18)
C10—Ru—C9	37.54 (8)	C11—C10—C9	122.7 (2)
C5—Ru—C9	161.32 (10)	C11—C10—Ru	72.98 (13)
C7—Ru—C9	68.15 (9)	C9—C10—Ru	72.34 (14)
C2—Ru—C11	172.26 (10)	C11—C10—H10	118.7
C8—Ru—C11	79.43 (8)	C9—C10—H10	118.7
C1—Ru—C11	134.63 (9)	Ru—C10—H10	128.4
C3—Ru—C11	142.82 (10)	C10—C11—C6	119.1 (2)
C4—Ru—C11	112.48 (10)	C10—C11—C14	119.8 (2)
C10—Ru—C11	37.37 (8)	C6—C11—C14	121.0 (2)
C5—Ru—C11	109.35 (9)	C10—C11—Ru	69.65 (12)
C7—Ru—C11	67.32 (8)	C6—C11—Ru	74.95 (12)
C9—Ru—C11	67.97 (9)	C14—C11—Ru	129.33 (16)
C2—Ru—C6	141.31 (11)	C7—C12—H12A	109.5
C8—Ru—C6	66.13 (8)	C7—C12—H12B	109.5
C1—Ru—C6	112.69 (9)	H12A—C12—H12B	109.5
C3—Ru—C6	173.84 (10)	C7—C12—H12C	109.5
C4—Ru—C6	136.54 (10)	H12A—C12—H12C	109.5
C10—Ru—C6	65.93 (9)	H12B—C12—H12C	109.5
C5—Ru—C6	110.54 (10)	C9—C13—H13A	109.5
C7—Ru—C6	36.71 (8)	C9—C13—H13B	109.5
C9—Ru—C6	78.77 (9)	H13A—C13—H13B	109.5
C11—Ru—C6	36.59 (8)	C9—C13—H13C	109.5
C5—C1—C2	108.5 (2)	H13A—C13—H13C	109.5
C5—C1—Ru	71.51 (14)	H13B—C13—H13C	109.5
C2—C1—Ru	70.50 (13)	C11—C14—H14A	109.5
C5—C1—H1	125.8	C11—C14—H14B	109.5
C2—C1—H1	125.8	H14A—C14—H14B	109.5
Ru—C1—H1	123.8	C11—C14—H14C	109.5
C1—C2—C3	108.0 (2)	H14A—C14—H14C	109.5
C1—C2—Ru	71.60 (14)	H14B—C14—H14C	109.5
C3—C2—Ru	71.60 (15)	F3—P—F2	91.16 (15)
C1—C2—H2	126.0	F3—P—F1	90.68 (13)
C3—C2—H2	126.0	F2—P—F1	178.11 (15)
Ru—C2—H2	122.5	F3—P—F6	90.80 (11)
C2—C3—C4	108.3 (2)	F2—P—F6	88.61 (11)
C2—C3—Ru	70.46 (15)	F1—P—F6	90.95 (10)
C4—C3—Ru	71.02 (17)	F3—P—F5	90.39 (11)
C2—C3—H3	125.8	F2—P—F5	90.78 (12)
C4—C3—H3	125.8	F1—P—F5	89.62 (11)
Ru—C3—H3	124.3	F6—P—F5	178.67 (11)
C3—C4—C5	107.1 (2)	F3—P—F4	178.94 (11)
C3—C4—Ru	70.88 (16)	F2—P—F4	89.55 (13)
C5—C4—Ru	71.14 (15)	F1—P—F4	88.61 (12)
C3—C4—H4	126.5	F6—P—F4	90.00 (10)
C5—C4—H4	126.5	F5—P—F4	88.81 (10)
Ru—C4—H4	123.2		
			
C2—Ru—C1—C5	−118.2 (2)	C9—Ru—C7—C8	−28.13 (13)
C8—Ru—C1—C5	170.50 (15)	C11—Ru—C7—C8	−102.44 (14)
C3—Ru—C1—C5	−80.34 (18)	C6—Ru—C7—C8	−128.9 (2)
C4—Ru—C1—C5	−37.51 (16)	C2—Ru—C7—C6	−145.36 (16)
C10—Ru—C1—C5	13.6 (5)	C8—Ru—C7—C6	128.9 (2)
C7—Ru—C1—C5	133.24 (16)	C1—Ru—C7—C6	−105.54 (16)
C9—Ru—C1—C5	−152.4 (2)	C3—Ru—C7—C6	−171.90 (18)
C11—Ru—C1—C5	58.7 (2)	C4—Ru—C7—C6	−65.4 (5)
C6—Ru—C1—C5	94.62 (17)	C10—Ru—C7—C6	63.41 (15)
C8—Ru—C1—C2	−71.3 (2)	C5—Ru—C7—C6	−70.65 (19)
C3—Ru—C1—C2	37.87 (17)	C9—Ru—C7—C6	100.76 (16)
C4—Ru—C1—C2	80.71 (19)	C11—Ru—C7—C6	26.45 (14)
C10—Ru—C1—C2	131.8 (4)	C2—Ru—C7—C12	−27.4 (3)
C5—Ru—C1—C2	118.2 (2)	C8—Ru—C7—C12	−113.1 (3)
C7—Ru—C1—C2	−108.54 (18)	C1—Ru—C7—C12	12.5 (2)
C9—Ru—C1—C2	−34.2 (3)	C3—Ru—C7—C12	−53.9 (3)
C11—Ru—C1—C2	176.94 (17)	C4—Ru—C7—C12	52.6 (5)
C6—Ru—C1—C2	−147.16 (17)	C10—Ru—C7—C12	−178.6 (2)
C5—C1—C2—C3	−1.0 (3)	C5—Ru—C7—C12	47.3 (3)
Ru—C1—C2—C3	−62.76 (18)	C9—Ru—C7—C12	−141.3 (2)
C5—C1—C2—Ru	61.77 (17)	C11—Ru—C7—C12	144.4 (2)
C8—Ru—C2—C1	125.65 (17)	C6—Ru—C7—C12	118.0 (3)
C3—Ru—C2—C1	−117.0 (2)	C6—C7—C8—C9	−4.4 (3)
C4—Ru—C2—C1	−79.74 (18)	C12—C7—C8—C9	177.4 (2)
C10—Ru—C2—C1	−161.60 (18)	Ru—C7—C8—C9	55.0 (2)
C5—Ru—C2—C1	−36.96 (16)	C6—C7—C8—Ru	−59.47 (19)
C7—Ru—C2—C1	86.09 (18)	C12—C7—C8—Ru	122.4 (2)
C9—Ru—C2—C1	163.97 (15)	C2—Ru—C8—C9	117.45 (16)
C6—Ru—C2—C1	53.2 (2)	C1—Ru—C8—C9	154.86 (14)
C8—Ru—C2—C3	−117.38 (16)	C3—Ru—C8—C9	78.45 (17)
C1—Ru—C2—C3	117.0 (2)	C4—Ru—C8—C9	53.6 (3)
C4—Ru—C2—C3	37.23 (17)	C10—Ru—C8—C9	−30.45 (14)
C10—Ru—C2—C3	−44.6 (3)	C5—Ru—C8—C9	172.9 (3)
C5—Ru—C2—C3	80.01 (18)	C7—Ru—C8—C9	−133.8 (2)
C7—Ru—C2—C3	−156.94 (16)	C11—Ru—C8—C9	−67.38 (14)
C9—Ru—C2—C3	−79.06 (18)	C6—Ru—C8—C9	−103.23 (15)
C6—Ru—C2—C3	170.13 (16)	C2—Ru—C8—C7	−108.73 (15)
C1—C2—C3—C4	1.5 (3)	C1—Ru—C8—C7	−71.33 (16)
Ru—C2—C3—C4	−61.3 (2)	C3—Ru—C8—C7	−147.74 (14)
C1—C2—C3—Ru	62.76 (17)	C4—Ru—C8—C7	−172.6 (2)
C8—Ru—C3—C2	78.66 (18)	C10—Ru—C8—C7	103.36 (15)
C1—Ru—C3—C2	−37.84 (16)	C5—Ru—C8—C7	−53.2 (3)
C4—Ru—C3—C2	−118.3 (2)	C9—Ru—C8—C7	133.8 (2)
C10—Ru—C3—C2	156.21 (15)	C11—Ru—C8—C7	66.43 (13)
C5—Ru—C3—C2	−80.13 (18)	C6—Ru—C8—C7	30.58 (13)
C7—Ru—C3—C2	41.4 (3)	C7—C8—C9—C10	−1.1 (4)
C9—Ru—C3—C2	117.05 (16)	Ru—C8—C9—C10	53.8 (2)
C11—Ru—C3—C2	−167.35 (16)	C7—C8—C9—C13	−178.8 (2)
C2—Ru—C3—C4	118.3 (2)	Ru—C8—C9—C13	−123.9 (2)
C8—Ru—C3—C4	−163.01 (16)	C7—C8—C9—Ru	−54.9 (2)
C1—Ru—C3—C4	80.49 (18)	C2—Ru—C9—C8	−78.26 (18)
C10—Ru—C3—C4	−85.46 (18)	C1—Ru—C9—C8	−54.8 (3)
C5—Ru—C3—C4	38.20 (17)	C3—Ru—C9—C8	−117.37 (16)
C7—Ru—C3—C4	159.71 (16)	C4—Ru—C9—C8	−155.31 (15)
C9—Ru—C3—C4	−124.62 (16)	C10—Ru—C9—C8	130.1 (2)
C11—Ru—C3—C4	−49.0 (2)	C5—Ru—C9—C8	−172.9 (3)
C2—C3—C4—C5	−1.4 (3)	C7—Ru—C9—C8	28.40 (13)
Ru—C3—C4—C5	−62.34 (18)	C11—Ru—C9—C8	101.79 (14)
C2—C3—C4—Ru	60.90 (19)	C6—Ru—C9—C8	65.17 (14)
C2—Ru—C4—C3	−37.07 (16)	C2—Ru—C9—C10	151.65 (16)
C8—Ru—C4—C3	36.3 (3)	C8—Ru—C9—C10	−130.1 (2)
C1—Ru—C4—C3	−79.55 (17)	C1—Ru—C9—C10	175.1 (2)
C10—Ru—C4—C3	110.69 (16)	C3—Ru—C9—C10	112.54 (17)
C5—Ru—C4—C3	−116.5 (2)	C4—Ru—C9—C10	74.59 (19)
C7—Ru—C4—C3	−123.1 (4)	C5—Ru—C9—C10	57.1 (4)
C9—Ru—C4—C3	72.48 (19)	C7—Ru—C9—C10	−101.70 (17)
C11—Ru—C4—C3	150.41 (16)	C11—Ru—C9—C10	−28.30 (15)
C6—Ru—C4—C3	−175.33 (16)	C6—Ru—C9—C10	−64.92 (16)
C2—Ru—C4—C5	79.45 (17)	C2—Ru—C9—C13	37.1 (3)
C8—Ru—C4—C5	152.8 (2)	C8—Ru—C9—C13	115.4 (3)
C1—Ru—C4—C5	36.97 (16)	C1—Ru—C9—C13	60.6 (3)
C3—Ru—C4—C5	116.5 (2)	C3—Ru—C9—C13	−2.0 (3)
C10—Ru—C4—C5	−132.79 (16)	C4—Ru—C9—C13	−39.9 (3)
C7—Ru—C4—C5	−6.6 (5)	C10—Ru—C9—C13	−114.5 (3)
C9—Ru—C4—C5	−171.01 (15)	C5—Ru—C9—C13	−57.5 (4)
C11—Ru—C4—C5	−93.07 (17)	C7—Ru—C9—C13	143.8 (2)
C6—Ru—C4—C5	−58.8 (2)	C11—Ru—C9—C13	−142.8 (3)
C2—C1—C5—C4	0.1 (3)	C6—Ru—C9—C13	−179.4 (2)
Ru—C1—C5—C4	61.23 (18)	C8—C9—C10—C11	1.7 (4)
C2—C1—C5—Ru	−61.13 (17)	C13—C9—C10—C11	179.4 (2)
C3—C4—C5—C1	0.8 (3)	Ru—C9—C10—C11	55.3 (2)
Ru—C4—C5—C1	−61.34 (17)	C8—C9—C10—Ru	−53.7 (2)
C3—C4—C5—Ru	62.16 (19)	C13—C9—C10—Ru	124.0 (2)
C2—Ru—C5—C1	37.26 (16)	C2—Ru—C10—C11	172.73 (19)
C8—Ru—C5—C1	−24.7 (4)	C8—Ru—C10—C11	−103.31 (14)
C3—Ru—C5—C1	79.91 (17)	C1—Ru—C10—C11	56.2 (5)
C4—Ru—C5—C1	118.1 (2)	C3—Ru—C10—C11	143.74 (15)
C10—Ru—C5—C1	−176.17 (15)	C4—Ru—C10—C11	103.37 (15)
C7—Ru—C5—C1	−63.83 (19)	C5—Ru—C10—C11	66.99 (18)
C9—Ru—C5—C1	141.8 (3)	C7—Ru—C10—C11	−66.08 (14)
C11—Ru—C5—C1	−139.86 (15)	C9—Ru—C10—C11	−133.6 (2)
C6—Ru—C5—C1	−100.86 (16)	C6—Ru—C10—C11	−30.24 (13)
C2—Ru—C5—C4	−80.81 (17)	C2—Ru—C10—C9	−53.7 (3)
C8—Ru—C5—C4	−142.8 (3)	C8—Ru—C10—C9	30.29 (15)
C1—Ru—C5—C4	−118.1 (2)	C1—Ru—C10—C9	−170.2 (4)
C3—Ru—C5—C4	−38.16 (16)	C3—Ru—C10—C9	−82.65 (18)
C10—Ru—C5—C4	65.76 (19)	C4—Ru—C10—C9	−123.03 (17)
C7—Ru—C5—C4	178.10 (15)	C5—Ru—C10—C9	−159.41 (16)
C9—Ru—C5—C4	23.8 (4)	C7—Ru—C10—C9	67.52 (16)
C11—Ru—C5—C4	102.07 (16)	C11—Ru—C10—C9	133.6 (2)
C6—Ru—C5—C4	141.07 (16)	C6—Ru—C10—C9	103.36 (17)
C2—Ru—C6—N	−56.5 (3)	C9—C10—C11—C6	3.4 (4)
C8—Ru—C6—N	−143.7 (3)	Ru—C10—C11—C6	58.46 (18)
C1—Ru—C6—N	−24.3 (3)	C9—C10—C11—C14	−179.6 (2)
C4—Ru—C6—N	50.5 (3)	Ru—C10—C11—C14	−124.5 (2)
C10—Ru—C6—N	142.1 (3)	C9—C10—C11—Ru	−55.0 (2)
C5—Ru—C6—N	16.2 (3)	N—C6—C11—C10	177.5 (2)
C7—Ru—C6—N	−112.3 (3)	C7—C6—C11—C10	−9.0 (3)
C9—Ru—C6—N	179.3 (3)	Ru—C6—C11—C10	−55.84 (18)
C11—Ru—C6—N	111.3 (3)	N—C6—C11—C14	0.5 (3)
C2—Ru—C6—C11	−167.79 (16)	C7—C6—C11—C14	174.0 (2)
C8—Ru—C6—C11	105.04 (14)	Ru—C6—C11—C14	127.2 (2)
C1—Ru—C6—C11	−135.59 (14)	N—C6—C11—Ru	−126.7 (2)
C4—Ru—C6—C11	−60.7 (2)	C7—C6—C11—Ru	46.86 (19)
C10—Ru—C6—C11	30.85 (13)	C8—Ru—C11—C10	65.58 (14)
C5—Ru—C6—C11	−95.09 (15)	C1—Ru—C11—C10	−165.34 (15)
C7—Ru—C6—C11	136.4 (2)	C3—Ru—C11—C10	−60.7 (2)
C9—Ru—C6—C11	68.03 (13)	C4—Ru—C11—C10	−90.98 (15)
C2—Ru—C6—C7	55.8 (2)	C5—Ru—C11—C10	−131.80 (15)
C8—Ru—C6—C7	−31.37 (15)	C7—Ru—C11—C10	102.99 (15)
C1—Ru—C6—C7	88.00 (17)	C9—Ru—C11—C10	28.42 (14)
C4—Ru—C6—C7	162.84 (15)	C6—Ru—C11—C10	129.52 (19)
C10—Ru—C6—C7	−105.56 (16)	C8—Ru—C11—C6	−63.94 (13)
C5—Ru—C6—C7	128.49 (16)	C1—Ru—C11—C6	65.14 (18)
C9—Ru—C6—C7	−68.38 (15)	C3—Ru—C11—C6	169.78 (16)
C11—Ru—C6—C7	−136.4 (2)	C4—Ru—C11—C6	139.50 (15)
N—C6—C7—C8	−177.1 (2)	C10—Ru—C11—C6	−129.52 (19)
C11—C6—C7—C8	9.5 (3)	C5—Ru—C11—C6	98.68 (15)
Ru—C6—C7—C8	56.62 (18)	C7—Ru—C11—C6	−26.53 (13)
N—C6—C7—C12	1.1 (4)	C9—Ru—C11—C6	−101.10 (14)
C11—C6—C7—C12	−172.4 (2)	C8—Ru—C11—C14	178.0 (2)
Ru—C6—C7—C12	−125.3 (2)	C1—Ru—C11—C14	−52.9 (3)
N—C6—C7—Ru	126.3 (2)	C3—Ru—C11—C14	51.7 (3)
C11—C6—C7—Ru	−47.15 (19)	C4—Ru—C11—C14	21.5 (2)
C2—Ru—C7—C8	85.75 (15)	C10—Ru—C11—C14	112.4 (3)
C1—Ru—C7—C8	125.57 (14)	C5—Ru—C11—C14	−19.3 (2)
C3—Ru—C7—C8	59.2 (2)	C7—Ru—C11—C14	−144.6 (2)
C4—Ru—C7—C8	165.7 (4)	C9—Ru—C11—C14	140.9 (2)
C10—Ru—C7—C8	−65.48 (14)	C6—Ru—C11—C14	−118.0 (3)
C5—Ru—C7—C8	160.46 (14)		

### Hydrogen-bond geometry (Å, °)

**Table:**

*D*—H···*A*	*D*—H	H···*A*	*D*···*A*	*D*—H···*A*
N—H1A···F1	0.87 (2)	2.26 (2)	3.106 (3)	163 (3)
N—H1B···F5^i^	0.87 (2)	2.43 (3)	3.174 (3)	143 (3)

Symmetry codes: (i) −*x*, *y*+1/2, −*z*+1/2.
